# Rapid urine-based screening for tuberculosis in HIV-positive patients admitted to hospital in Africa (STAMP): a pragmatic, multicentre, parallel-group, double-blind, randomised controlled trial

**DOI:** 10.1016/S0140-6736(18)31267-4

**Published:** 2018-07-28

**Authors:** Ankur Gupta-Wright, Elizabeth L Corbett, Joep J van Oosterhout, Douglas Wilson, Daniel Grint, Melanie Alufandika-Moyo, Jurgens A Peters, Lingstone Chiume, Clare Flach, Stephen D Lawn, Katherine Fielding

**Affiliations:** a TB Centre, London School of Hygiene & Tropical Medicine, London, UK; b Department of Clinical Research, Faculty of Infectious and Tropical Diseases, London School of Hygiene & Tropical Medicine, London, UK; c Department of Infectious Disease Epidemiology, Faculty of Epidemiology and Population Health, London School of Hygiene & Tropical Medicine, London, UK; d Malawi-Liverpool-Wellcome Trust Clinical Research Programme, College of Medicine, Blantyre, Malawi; e Dignitas International, Zomba, Malawi; f College of Medicine, University of Malawi, Blantyre, Malawi; g Department of Medicine, Edendale Hospital, University of KwaZulu-Natal, Pietermaritzburg, South Africa; h Division of Health and Social Care Research, Faculty of Life Sciences and Medicine, Kings College London, London, UK; i School of Public Health, Faculty of Health Sciences, University of the Witwatersrand, Johannesburg, South Africa

## Abstract

**Background:**

Current diagnostics for HIV-associated tuberculosis are suboptimal, with missed diagnoses contributing to high hospital mortality and approximately 374 000 annual HIV-positive deaths globally. Urine-based assays have a good diagnostic yield; therefore, we aimed to assess whether urine-based screening in HIV-positive inpatients for tuberculosis improved outcomes.

**Methods:**

We did a pragmatic, multicentre, double-blind, randomised controlled trial in two hospitals in Malawi and South Africa. We included HIV-positive medical inpatients aged 18 years or more who were not taking tuberculosis treatment. We randomly assigned patients (1:1), using a computer-generated list of random block size stratified by site, to either the standard-of-care or the intervention screening group, irrespective of symptoms or clinical presentation. Attending clinicians made decisions about care; and patients, clinicians, and the study team were masked to the group allocation. In both groups, sputum was tested using the Xpert MTB/RIF assay (Xpert; Cepheid, Sunnyvale, CA, USA). In the standard-of-care group, urine samples were not tested for tuberculosis. In the intervention group, urine was tested with the Alere Determine TB-LAM Ag (TB-LAM; Alere, Waltham, MA, USA), and Xpert assays. The primary outcome was all-cause 56-day mortality. Subgroup analyses for the primary outcome were prespecified based on baseline CD4 count, haemoglobin, clinical suspicion for tuberculosis; and by study site and calendar time. We used an intention-to-treat principle for our analyses. This trial is registered with the ISRCTN registry, number ISRCTN71603869.

**Findings:**

Between Oct 26, 2015, and Sept 19, 2017, we screened 4788 HIV-positive adults, of which 2600 (54%) were randomly assigned to the study groups (n=1300 for each group). 13 patients were excluded after randomisation from analysis in each group, leaving 2574 in the final intention-to-treat analysis (n=1287 in each group). At admission, 1861 patients were taking antiretroviral therapy and median CD4 count was 227 cells per μL (IQR 79–436). Mortality at 56 days was reported for 272 (21%) of 1287 patients in the standard-of-care group and 235 (18%) of 1287 in the intervention group (adjusted risk reduction [aRD] −2·8%, 95% CI −5·8 to 0·3; p=0·074). In three of the 12 prespecified, but underpowered subgroups, mortality was lower in the intervention group than in the standard-of-care group for CD4 counts less than 100 cells per μL (aRD −7·1%, 95% CI −13·7 to −0·4; p=0.036), severe anaemia (−9·0%, −16·6 to −1·3; p=0·021), and patients with clinically suspected tuberculosis (−5·7%, −10·9 to −0·5; p=0·033); with no difference by site or calendar period. Adverse events were similar in both groups.

**Interpretation:**

Urine-based tuberculosis screening did not reduce overall mortality in all HIV-positive inpatients, but might benefit some high-risk subgroups. Implementation could contribute towards global targets to reduce tuberculosis mortality.

**Funding:**

Joint Global Health Trials Scheme of the Medical Research Council, the UK Department for International Development, and the Wellcome Trust.

## Introduction

Tuberculosis remains the single major cause of mortality in patients with HIV globally, accounting for an estimated 374 000 deaths in 2016.^1^ In many parts of sub-Saharan Africa, most admitted medical inpatients are HIV-positive and tuberculosis is the major cause of both admission (18–29%) and in-hospital death (21–33% in cohort studies and 32–67% in autopsy studies).^2^, ^3^

Suboptimal diagnostics are an important contributor to poor outcomes from HIV-associated tuberculosis. Tuberculosis is commonly disseminated, presents with non-specific clinical features, and is only diagnosed before death in half of cases with a fatal outcome.^3^, ^4^ Mycobacterial culture, the current gold standard, is too centralised and slow to be clinically useful. Both culture and chest radiography are often unavailable in many African settings. The Xpert MTB/RIF assay provides robust and rapid detection of *Mycobacterium tuberculosis* nucleic acids from sputum and has been widely scaled-up and decentralised, but patients with HIV-associated tuberculosis tend to have relatively low mycobacterial concentrations in pulmonary secretions and difficulty expectorating.^5^ Despite improved sensitivity (79% in patients with HIV),^6^ randomised trials comparing clinical outcomes between sputum Xpert MTB/RIF and microscopy have largely shown scant effect because of empiric tuberculosis therapy, other than systematic screening in HIV-positive outpatients with advanced disease.^7^, ^8^

**Research in context**

**Evidence before the study**

We searched MEDLINE for studies that investigated the effect of urine lipoarabinomannan assay (LAM) or Xpert MTB/RIF assay (Xpert) on mortality or tuberculosis diagnosis in HIV-positive patients published from Jan 1, 2000, to Sept 30, 2016. We combined search terms for LAM (“lipoarabinomannan”, “LAM”, “TB LAM”, or “urine LAM”) or Xpert (“urine Xpert” or “urinary Xpert”) with HIV (“HIV”, “HIV-1”, “AIDS”, or “human immunodeficiency virus”) and mortality (“mortality”, “adult mortality”, or “death”), or tuberculosis diagnosis or screening (“diagnosis”, “diagnostic”, or “screening”). We identified 14 observational studies, mostly done in antiretroviral therapy naive outpatients or hospital inpatients, which assessed the diagnostic accuracy of urine LAM or Xpert for tuberculosis or their association with mortality, or both. These studies showed moderate-to-good diagnostic yield of urinary assays in patients with advanced immunosuppression and in hospital inpatients, and an association with higher disease severity, poor prognosis, and mortality. Since undertaking our trial, one randomised trial has assessed adjunctive urine LAM testing in HIV-positive inpatients suspected of tuberculosis and found a reduction in 8-week mortality. However, whether systematic urine-based screening for tuberculosis (using urine LAM and Xpert) for all HIV-positive hospital inpatients (irrespective of tuberculosis symptoms) could reduce mortality compared with sputum tuberculosis testing remained unclear.

**Added value of this study**

The findings from this randomised trial suggest that urine-based tuberculosis screening of HIV-positive hospital inpatients might reduce 56-day mortality in defined clinical subgroups (low CD4 count, severe anaemia, or clinically suspected tuberculosis). Moreover, wider application (screening all HIV-positive inpatients) could substantially reduce the risk of being discharged from hospital with undiagnosed tuberculosis in all patient groups. The major incremental diagnostic benefit was from urine LAM.

**Implications of all the available evidence**

These data support implementation of urine LAM-based screening of all HIV-positive inpatients for tuberculosis in hospitals in high HIV and tuberculosis burden settings, because the reliance on a combination of sputum-based diagnosis and clinically guided empirical treatment left patients at unacceptably high risk of discharge and death from undiagnosed tuberculosis. Collectively, current evidence supports international policy change to scale-up and broaden the use of urine-LAM testing in patients admitted to hospital (currently only recommended as an additional diagnostic in inpatients with symptoms of tuberculosis and CD4 counts <100 cells per μL or danger signs). Incremental gain was too limited to support urine Xpert. Because screening efficiency is dependent on prevalence, these results cannot be extrapolated to outpatients. Urine LAM screening could contribute towards reducing mortality and morbidity from HIV-associated tuberculosis and meeting global targets for tuberculosis mortality reduction.

Urine can be readily obtained from patients admitted to hospital and is suitable for rapid tuberculosis diagnosis using either a lateral flow assay for lipoarabinomannan (LAM; a mycobacterial cell wall glycolipid) or Xpert MTB/RIF. Although urine is not a sample recommended by WHO for Xpert, studies report high specificity for tuberculosis in HIV-positive patients.^9^, ^10^ Diagnosis using urinary LAM, reflecting frequent renal involvement from disseminated HIV-associated tuberculosis, is complementary to sputum testing, and identifies a subgroup of patients with poor prognosis.^11^, ^12^ Current commercial LAM kits have a specificity of 98% or more and sensitivity of 40–70% in HIV–tuberculosis-coinfected patients with CD4 counts less than 100 cells per μL.^13^, ^14^, ^15^ Combined testing with urine LAM, plus urine and sputum Xpert, can rapidly diagnose about 80% of all culture-positive tuberculosis in unselected HIV-positive admissions to medical wards in high HIV burden settings.^15^, ^16^

Urine-based screening might provide more complete, timely, and potentially life-saving diagnosis of tuberculosis among HIV-positive hospital inpatients.^7^ We, therefore, aimed to investigate the effect of adding urine to sputum tuberculosis screening on early mortality, and its effect on diagnosis and treatment of tuberculosis in unselected HIV-positive hospital admissions.

## Methods

### Study design and patients

We did a pragmatic, multicentre, parallel-group, double-blind, randomised controlled trial. We enrolled patients who were admitted to medical wards at Zomba Central Hospital in Malawi (a district and referral hospital covering urban and rural populations) and Edendale Hospital in South Africa (a large referral hospital covering a mostly urban population), irrespective of tuberculosis symptoms or admitting presentation. The study design has been previously described in detail,^17^ and additional methods are provided in the appendix. We obtained ethical approval from the relevant committees in Malawi, South Africa, and from the trial sponsor in the UK, and the study was approved by the relevant national regulatory bodies (appendix). Deviations from the study protocol are described in the appendix.

All admissions to the medical wards were screened for eligibility by study nurses or clinicians. Screening occurred during office hours on weekdays, with patients enrolled as close to admission as possible. All patients with an unknown HIV status were offered point-of-care rapid HIV testing as per local guidelines (appendix). We included patients that were HIV positive and aged 18 years or older. We excluded those that were currently taking tuberculosis treatment, had been treated for tuberculosis in the preceding 12 months, taken isoniazid preventive therapy in the preceding 6 months, were unable or unwilling to provide informed consent, had been admitted to hospital for longer than 48 h at the time of screening, or lived outside the predefined hospital catchment area (appendix). We obtained written informed consent from all eligible patients.

### Randomisation and masking

We randomly assigned eligible patients on enrolment (1:1) to either the standard-of-care tuberculosis screening group or the intervention screening group. Randomisation was stratified by site and a randomisation list of unique patient identifiers was generated by the study statistician using a computer-generated random block size. On enrolment, study nurses or clinicians took a consecutive sealed opaque envelope containing the unique patient identifier but not the study group, to which they remained masked. A paired set of sealed envelopes were kept in a locked cabinet in the study laboratory, labelled with the unique patient identifier and containing the study group allocation. These were opened by the laboratory technician on receipt of study tuberculosis screening specimens. Investigators, all study staff (other than the laboratory technician and statistician), hospital attending clinical teams, and patients were masked to the study group allocation.

### Procedures

Following enrolment, 50 mL of urine and a single, spontaneously expectorated sputum sample were collected by the study team for tuberculosis screening. Failure to produce a specimen was not an exclusion criterion. The patient's attending clinical team had the option of sending additional samples for routine tuberculosis investigations available at the study hospital; the appendix provides further details of the tests available at each hospital.

Tuberculosis screening samples (ie, sputum or urine, or both) were processed according to study group allocation by the study laboratory technician, and assays were run during office hours and processed as soon as possible after arrival of a specimen in the laboratory. In both groups, sputum was tested using the Xpert MTB/RIF assay (Xpert; Cepheid, Sunnyvale, CA, USA). In the standard-of-care group, urine samples were not tested for tuberculosis. In the intervention group, 60 μL of unconcentrated urine was tested with the Alere Determine TB-LAM Ag assay (TB-LAM; Alere, Waltham, MA, USA) as per the manufacturer's instructions, and 40–50 mL of urine was concentrated by centrifugation for testing with Xpert. Urine Xpert and TB-LAM were processed simultaneously. We deemed TB-LAM positive using the grade 1 cutoff on the manufacturer's post-2014 reference card, which was referred to as the grade 2 cutoff before 2014. The appendix provides further details of the laboratory methods used in this study.

Once all the tuberculosis specimens received had been processed, tuberculosis screening results were reported to the attending clinical team as positive, negative, or not done to maintain masking, with neither study group nor individual test results communicated to attending clinical or study teams. Rifampicin resistance results, if available, were also reported (appendix). Clinical management, including tuberculosis treatment decisions and management of antiretroviral therapy (ART), relied on the attending clinical team according to local and national guidelines and was independent of study nurses, clinicians, or investigators.

The study team documented patients' clinical events during hospital admission, including but not limited to tuberculosis investigations and diagnosis, commencement of tuberculosis treatment and any side-effects, management of HIV (including stopping or starting ART), and discharge or death. Follow-up at 56 days for those discharged from hospital alive was done in person by outpatient attendance. Those who did not attend were contacted by telephone or a home visit, or both, with interview of next of kin to establish vital status if required.

### Outcomes

The primary outcome was the cumulative risk of all-cause mortality at 56 days from enrolment. Subgroup analysis for the primary outcome was prespecified in populations with higher risk of tuberculosis, mortality, or both (ie, low baseline CD4 cell count, low haemoglobin, or clinical suspicion for tuberculosis); and by study site and calendar time. Secondary outcomes were time to mortality, proportions of patients with microbiologically confirmed tuberculosis and clinically diagnosed tuberculosis, time from randomisation to tuberculosis diagnosis and to start of tuberculosis treatment, time from tuberculosis diagnosis to treatment initiation, prescription of antimicrobials, ART initiation (if ART naive at hospital admission), duration of hospitalisation, and hospital readmission events.

Microbiologically confirmed tuberculosis was defined as one or more positive specimens for acid fast bacilli, Xpert, mycobacterial culture, or TB-LAM. Clinically diagnosed tuberculosis was defined by the decision to treat for tuberculosis in the absence of microbiological confirmation. Patients with any tuberculosis diagnosis (microbiologically confirmed or clinically diagnosed) were also reported. We recorded for all patients whether tuberculosis was included in the admitting differential diagnoses by attending clinicians, referred to as clinically suspected tuberculosis.

### Statistical analysis

The sample size calculation was based on the assumption that 56-day mortality risk would be 25% in the standard-of-care group, and loss to follow-up would be 10% or less. We therefore calculated that enrolling 1300 patients per group would provide at least 80% power to detect a 5% absolute mortality reduction in the intervention group, with a two-sided type 1 error of 5% (appendix).

We used an intention-to-treat principle for all our analyses, including all eligible patients that were randomly assigned. For the primary outcome, we calculated a risk difference with 95% CIs for mortality at 56 days comparing the standard-of-care group with the intervention group with the following: using a generalised linear model with identity link function and binomial family, adjusting for study site, using a fixed effect, and assuming participants lost to follow-up had not died. An odds ratio adjusted for site with 95% CIs was also calculated using logistic regression. Prespecified subgroup analyses were done for the primary outcome. These subgroups were study site (Malawi or South Africa), baseline CD4 counts (<100 cells per μL or ≥100 cells per μL), severe anaemia (haemoglobin <8 g/dL or ≥8 g/dL), whether tuberculosis was clinically suspected at admission, and calendar time (by 6 monthly intervals from Oct 1, 2015, to Sep 30, 2017). A sensitivity analysis was also done assuming all losses to follow-up had died.

Secondary outcomes were compared between the study groups using adjusted risk difference and adjusted odds ratio (aOR) for binary outcomes, Cox proportional hazards regression for time-to-event outcomes, and Kaplan-Meier curves for time to mortality. 95% CI were calculated for all analyses. In exploratory post-hoc analyses, tuberculosis diagnoses were also compared between study groups using the same subgroups as the primary outcome (study site, baseline CD4 cell count, severe anaemia, and clinically suspected tuberculosis) to investigate whether the absence of mortality benefit was accompanied by a lack of difference in tuberculosis diagnosis. Diagnostic yields of urine-based tuberculosis tests were calculated post hoc as a proportion of all microbiologically confirmed tuberculosis to better understand the respective contributions of TB-LAM and urine Xpert.

We did all the analyses using SAS (version 9.4). This study is registered with the ISRCTN registry, number ISRCTN71603869.

### Role of the funding source

The funder of the study had no role in study design, data collection, data analysis, data interpretation, or writing of the report. The corresponding author had full access to all the data in the study and had final responsibility for the decision to submit for publication.

## Results

**Figure 1:**
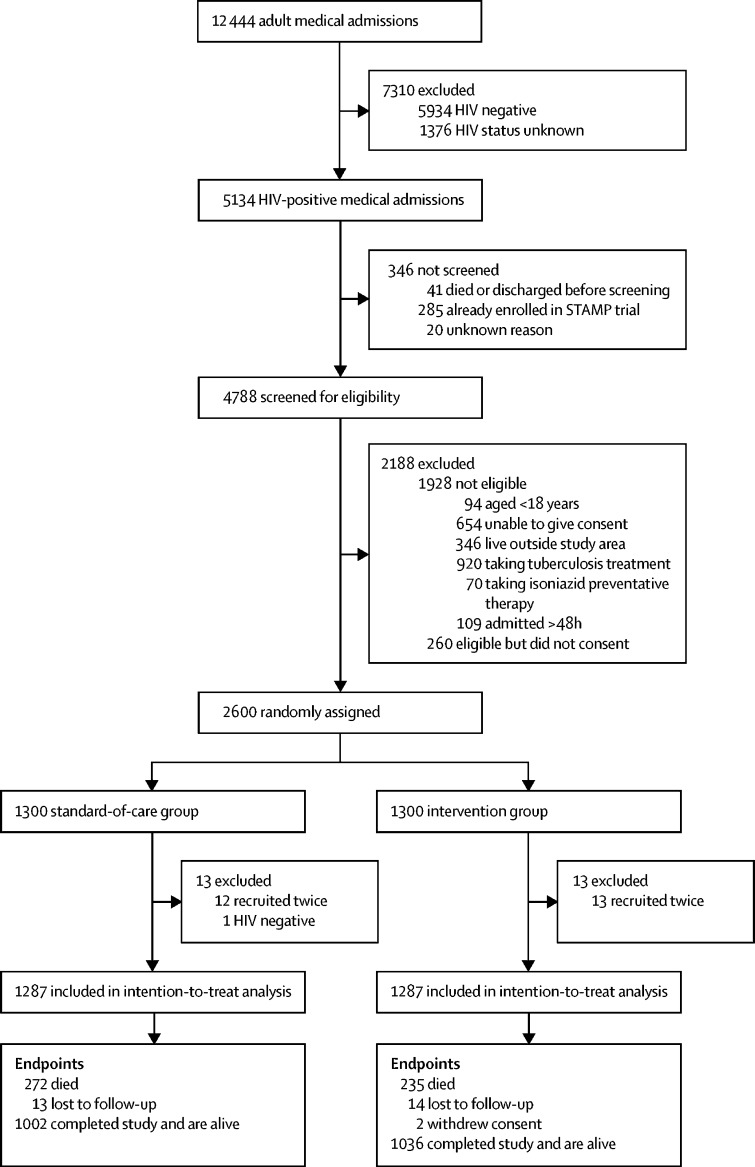
Trial profile Patients could have more than one reason for exclusion. Data stratified by site are shown in the appendix.

Between Oct 26, 2015, and Sept 19, 2017, we screened 4788 HIV-positive adult medical admissions. Of these admissions, 1928 (40%) patients were ineligible, 260 (5%) did not provide consent to participate, and 2600 (54%) were randomly assigned to the study groups (n=1300 for each group; figure 1). The appendix shows those who were excluded by site. 26 patients were excluded after randomisation because of ineligibility, leaving 2574 in the final intention-to-treat analysis (n=1287 in each group). 27 (1%) of 2574 patients were lost to follow-up at 56 days after hospital discharge (figure 1).

Baseline characteristics were balanced between the study groups (table; appendix). Mean age was 39·6 years (SD 11·7 years) and 1461 (57%) of 2574 participants were women. 2168 (84%) patients already knew their HIV diagnosis before admission, of whom 1861 (86%) were currently taking ART. Median CD4 count was 227 cells per μL (IQR 79–436), 748 (29%) of 2574 patients had a CD4 count of less than 100 cells per μL, and 587 (23%) had severe anaemia (haemoglobin <8 g/dL). 1332 (52%) patients reported a cough, 2316 (90%) had one or more WHO tuberculosis symptoms (ie, cough, fever, weight loss, or night sweats), and 996 (39%) were clinically suspected of tuberculosis at admission. Differences between sites included higher ART coverage, fewer patients reporting cough, fewer able to expectorate sputum, and fewer having clinically suspected tuberculosis at admission in Malawi than in South Africa (table).

**Table:** Patient characteristics at enrolment by study group and country

			**Standard-of-care group (n=1287)**	**Intervention group (n=1287)**	**Malawi (n=1316)**	**South Africa (n=1258)**
Age (years)			39·6 (11·9)	39·7 (11·6)	40·1 (11·7)	39·1 (11·7)
Sex						
	Women		734 (57%)	727 (56%)	829 (63%)	632 (50%)
	Men		553 (43%)	560 (44%)	487 (37%)	626 (50%)
	New HIV diagnosis		212 (16%)	194 (15%)	208 (16%)	198 (16%)
ART status*						
	Never		93 (9%)	121 (11%)	57 (5%)	157 (15%)
	Currently taking		935 (87%)	926 (85%)	1021 (92%)	840 (79%)
	Interrupted		47 (4%)	46 (4%)	30 (3%)	63 (6%)
Time on ART (years)†			3·0 (0·7–6·7)	3·0 (0·8–6·7)	3·4 (0·8–7·4)	2·6 (0·6–5·8)
TB symptoms reported						
	Cough		681 (53%)	651 (51%)	611 (46%)	721 (57%)
	Fever		747 (58%)	753 (59%)	761 (58%)	739 (59%)
	Night sweats‡		540 (42%)	497 (39%)	488 (37%)	549 (44%)
	Weight loss‡		875 (68%)	906 (70%)	863 (66%)	918 (73%)
Any WHO TB symptom			1164 (90%)	1152 (90%)	1187 (90%)	1129 (90%)
Clinically suspected TB§			495 (38%)	501 (39%)	353 (27%)	643 (51%)
Previous TB treatment			309 (24%)	335 (26%)	202 (15%)	442 (35%)
Body-mass index (kg/m^2^)			21·7 (5·8)	21·6 (5·8)	20·0 (4·1)	23·3 (6·7)
Morbidity at admission						
	WHO danger sign¶		275 (21%)	277 (22%)	337 (26%)	215 (17%)
	Karnofsky score		60 (50–70)	60 (50–70)	60 (50–70)	60 (50–70)
	CD4 cell count§					
		Median (cells per μL)	222 (80–436)	231 (78–438)	219 (86–431)	236 (70–445)
		<100 cells per μL	377 (29%)	371 (29%)	365 (28%)	383 (30%)
	Haemoglobin‖					
		Median (g/dL)	10·4 (8·1–12·9)	10·8 (8·3–12·7)	10·4 (7·8–12·4)	113 (8·8–13·1)
		<8 g/dL	298 (23%)	289 (22%)	355 (27%)	232 (18%)

Data are mean (SD), n (%), or median (IQR). TB symptoms are reported if present for any duration. ART=antiretroviral therapy. TB=tuberculosis.

* ART status denominator is the number of patients with a known HIV diagnosis (n=1075 for standard-of-care group and n=1093 for intervention group).

† Missing data for 26 patients in Malawi and 26 in South Africa.

‡ Missing data for one patient in South Africa.

§ Missing data for three patients in Malawi and six in South Africa.

¶ WHO danger signs are one or more of the following: heart rate more than 120 beats per minute, respiratory rate more than 30 breaths per minute, temperature more than 39°C, or being unable to walk unaided.

‖ Missing data for five patients in South Africa.

**Figure 2:**
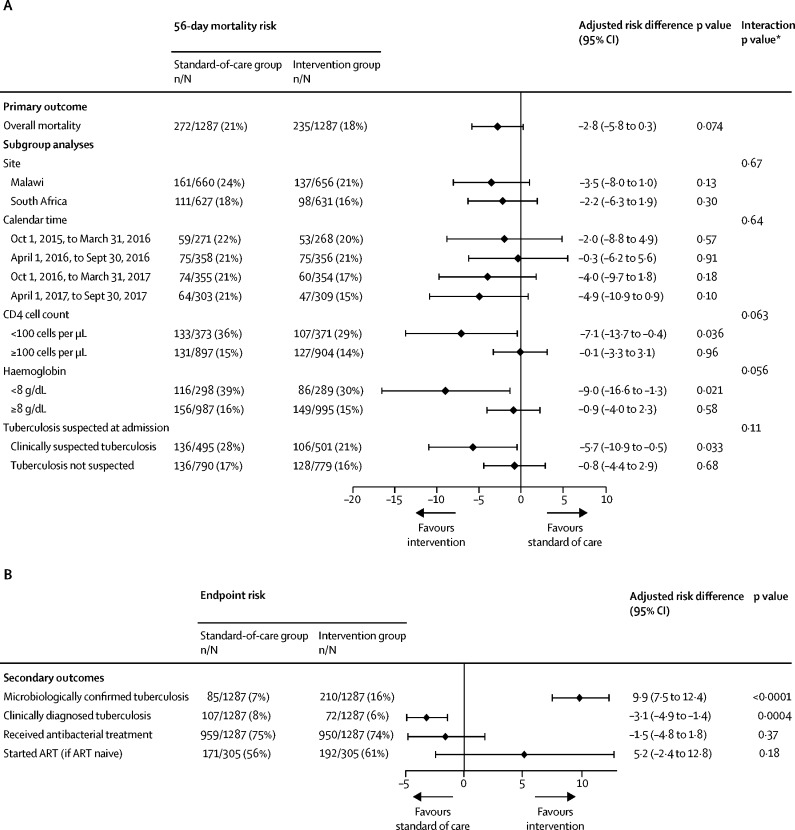
Primary outcome and predefined subgroup analyses (A), and secondary outcomes (B) All analyses are adjusted for study site. (A) The primary outcome is mortality at 56 days after randomisation. Risk differences are the risk in the intervention group minus the risk in the standard-of-care group. (B) Secondary outcomes are measured at the end of hospital admission except for those who started ART, which is measured at 56 days. Antibacterial treatment excludes anti-TB medications. ART=antiretroviral therapy. *Interaction between study group and subgroup.

By 56 days, 507 (20%) of 2574 patients had died: 272 (21%) of 1287 in the standard-of-care group and 235 (18%) of 1287 in the intervention group, giving an adjusted risk difference of −2·8% (95% CI −5·8 to 0·3; p=0·074; figure 2). The aOR for mortality in the intervention group compared with the standard-of-care group was 0·83 (95% CI 0·69–1·01; p=0·068; appendix). Mortality in the intervention group was significantly lower than in the standard-of-care group for the three prespecified high-risk subgroups: adjusted risk difference −7·1% (95% CI −13·7 to −0·4) in patients with baseline CD4 counts less than 100 cells per μL, −9·0% (−16·6 to −1·3) in patients with severe anaemia, and −5·7% (−10·9 to −0·5) in patients with clinically suspected tuberculosis at admission (figure 2). p values for interaction between the subgroup and study group are reported in figure 2. 1567 (61%) of 2574 patients were in one or more high-risk subgroups (low CD4 count, severe anaemia, or clinically suspected tuberculosis). Sensitivity analysis assuming losses to follow-up had died did not alter overall or subgroup mortality risk differences (appendix).

**Figure 3:**
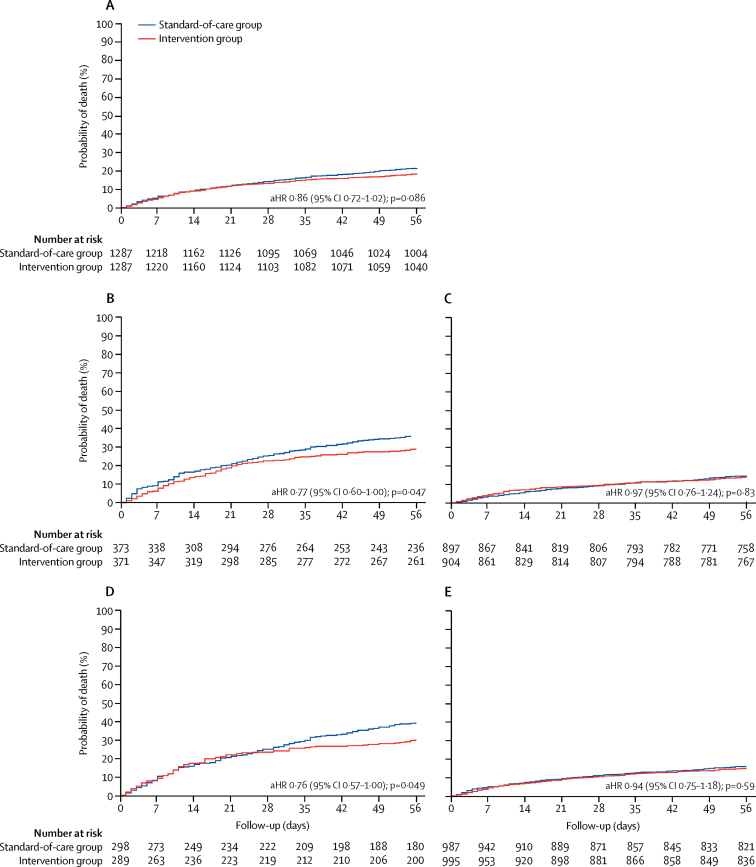
Time to 56-day mortality overall and stratified by high-risk subgroups All aHRs were adjusted for study site. (A) Survival analysis over 56 days in the standard-of-care group and intervention group. (B) Survival analysis stratified by CD4 counts less than 100 cells per μL in both groups. (C) Survival analysis stratified by CD4 counts of 100 cells per μL or more in both groups. (D) Survival analysis stratified by haemoglobin of less than 8 g/dL in both groups. (E) Survival analysis stratified by haemoglobin of 8 g/dL or more in both groups. aHR=adjusted hazard ratio.

Overall, 36 patients would need to be screened with the study intervention (ie, TB-LAM and urine Xpert) to prevent one death (appendix). Median duration of hospital stay was 6 days (IQR 2–11), and did not differ between the two groups (appendix). Although 273 (54%) of 507 deaths occurred during hospital admission, overall and high-risk subgroup survival curves did not substantially diverge until after day 21 (figure 3). Among patients discharged alive from hospital, 134 (12%) of 1146 patients died in the standard-of-care group and 100 (9%) of 1150 died in the intervention group. In time-to-mortality analysis, the adjusted hazard ratio (aHR) for intervention compared with standard of care was 0·86 (95% CI 0·72–1·02; p=0·086; figure 3A).

Of the study's tuberculosis screening samples at baseline, urine was provided by 2548 (99%) of 2574 patients, whereas only 1464 (57%) produced sputum (518 [39%] of 1316 in Malawi and 946 [75%] of 1258 in South Africa). Chest radiographs as part of routine care were taken in 1231 (48%) of 2574 patients during inpatient stay (300 [23%] of 1316 in Malawi and 931 [74%] of 1258 in South Africa; appendix). Overall, tuberculosis was diagnosed during hospital admission in 474 (18%) of 2574 patients, with 282 (22%) diagnoses in the intervention group and 192 (15%) in the standard-of-care group (appendix). The adjusted risk difference for tuberculosis diagnosis between the two groups was 7·3% (95% CI 4·4–10·2; p<0·0001). The intervention group also had more microbiologically confirmed tuberculosis diagnoses than the standard-of-care group (210 [16%] of 1287 *vs* 85 [7%] of 1287; adjusted risk difference 9·9% [95% CI 7·5–12·4]; p<0·0001) and fewer clinically diagnosed tuberculosis (77 [6%] *vs* 114 [9%]; adjusted risk difference −3·1% [–4·9 to −1·4]; p=0·0004; figure 2). 14 patients would need to be screened with the study intervention to prevent one missed tuberculosis diagnosis (appendix).

Time from randomisation to tuberculosis diagnosis was marginally shorter in the intervention group than in the standard-of-care group (median 0 days [IQR 0–1] *vs* 1 day [0–6]; appendix). 450 patients were started on tuberculosis treatment during admission, 268 in the intervention group and 182 in the standard-of-care group (aHR 1·56, 95% CI 1·29–1·88; p<0·0001; appendix). Time from diagnosis to tuberculosis treatment was universally short (median of 1 day, IQR 0–1) and was similar in both groups. Adverse events related to tuberculosis treatment were similar in both groups; the appendix summarises these adverse events. Antibacterial treatment and ART initiation did not differ by group (figure 2), although time to ART initiation was shorter in the intervention group than in the standard-of-care group (appendix). Of the 24 patients diagnosed with tuberculosis but not started on treatment during hospital admission, ten (42%) had died and 14 (58%) had been prematurely discharged. Only 27 other patients were started on tuberculosis treatment between discharge and day 56, with no difference between groups (appendix). Hospital readmission, losses to follow-up, adverse events (tuberculosis treatment discontinuation and side-effects), and rifampicin resistance detection did not differ between groups (appendix).

In post-hoc exploratory analyses, the increases in tuberculosis diagnoses in the intervention group versus the standard-of-care group were not confined to high-risk subgroups, unlike mortality, with an adjusted absolute risk increase of 7·0% (95% CI 4·1–10·0) in tuberculosis diagnoses in patients with CD4 counts of 100 cells per μL or more, and 8·0% (5·0–11·1) in those not clinically suspected of tuberculosis at admission (appendix). Increased tuberculosis diagnoses were more pronounced in Malawi than South Africa, although there was no evidence for an interaction between group and country (p=0·19). The largest increase in tuberculosis diagnoses in the intervention group was in the severe anaemia group, with an adjusted risk increase of 18·6% (95% CI 11·5–25·6; interaction p=0·0002). There were 93 extra patients on treatment for confirmed tuberculosis who were discharged alive and 34 fewer post-discharge deaths in the intervention group than in the standard-of-care group. Sputum Xpert diagnosed a similar number of patients with tuberculosis in both groups (appendix). In the intervention group, TB-LAM provided the highest diagnostic yield (158 [75%] of 210 patients with microbiologically confirmed tuberculosis), compared with 74 (35%) patients positive with urine Xpert and 85 (40%) positive with sputum Xpert (appendix). The incremental diagnostic yield from urine Xpert as the only positive assay was only 13 (6%) patients, compared with 87 (41%) patients from TB-LAM and 30 (14%) patients from sputum Xpert.

## Discussion

Although the 56-day all-cause mortality showed no significant differences between the standard-of-care and intervention groups, the addition of urine-based tuberculosis screening using TB-LAM and Xpert to sputum-based screening in all HIV-positive medical inpatients significantly reduced mortality at 56 days in prespecified high-risk subgroups, and substantially increased tuberculosis diagnoses and treatment across all patients. Fewer patients were on tuberculosis treatment at discharge in the standard-of-care group than in the intervention group, and more patients died after discharge, suggesting discharge with undiagnosed and untreated active tuberculosis as the main underlying mechanism. For every ten extra patients with confirmed tuberculosis discharged on treatment in the intervention group, there were 3·7 fewer deaths after discharge, supporting high individual risk of rapid progression to death in undiagnosed HIV-associated tuberculosis that could have been detected and treated through urine-based screening.

Morbidity and mortality from HIV-associated tuberculosis are slowly decreasing in Africa, mainly reflecting the expansion of HIV diagnosis and ART programmes rather than tuberculosis-specific diagnostic and prevention interventions.^18^, ^19^ Although these trends are encouraging, we found disturbingly high risk of death or microbiologically confirmed tuberculosis, or both, within 56 days of admission, despite high ART coverage and median CD4 cell count. We report substantial mortality reductions from urine-based tuberculosis screening in prespecified high-risk subgroups, consistent with current recommendations for diagnostic LAM testing, but no significant effect on overall mortality at 56 days between groups (adjusted risk difference −2·8%, 95% CI −5·8 to 0·3). However, our study was underpowered to detect small (<5%) absolute reductions in mortality at 56 days.

Our findings are consistent with the 4% (95% CI 1–7) mortality reduction in HIV-positive inpatients with clinically suspected tuberculosis reported from a diagnostic (not screening) randomised trial of urine LAM testing.^20^ The participant profile in the diagnostic trial differed notably from this study, with lower ART coverage and CD4 counts (median 84 cells per μL *vs* 227 cells per μL), and a greater proportion of participants had tuberculosis (intervention groups: 51·6% *vs* 21·8%), reflecting different inclusion criteria (clinical suspicion of tuberculosis compared with an unselected population in our STAMP trial), as well as underlying population trends in ART coverage. Early survival benefit from these two trials underscores the fulminant course of undiagnosed tuberculosis in highly immunosuppressed patients, and the higher yield of urinary diagnostics and difficulty diagnosing tuberculosis among groups of hospitalised HIV-positive patients by other means.^13^, ^15^

Uniquely, STAMP recruited considerable numbers of less immunosuppressed or critically ill patients who fall outside current recommendations for urinary tuberculosis diagnostic assays.^21^ We show differences between groups in tuberculosis diagnosis, although with a corresponding mortality benefit only for predefined high-risk groups (ie, low CD4 cell counts, low haemoglobin, or clinically suspected tuberculosis). The absence of detectable survival benefit in patients with less profound immunosuppression might then simply reflect a slower time-course if median survival following discharge with undiagnosed tuberculosis is considerably longer than 56 days. If so, increases in tuberculosis treatment through early urine-based diagnosis will still have averted months of morbidity and contributed to reduced transmission, particularly in health-care settings.^22^ An alternative explanation is a higher proportion of false-positive urinary screening results in patients with CD4 counts of 100 cells per μL or more, which we consider unlikely given high specificity (≥99%) shown elsewhere.^14^, ^15^, ^23^

Better clinical acumen and alternative investigations such as radiology leading to early empirical tuberculosis treatment can mitigate the effect of new diagnostic tests, as observed in relatively well resourced inpatient and outpatient settings, for example in South Africa.^24^ We saw little evidence of this effect for urine-based screening in either Malawi or South Africa in our STAMP trial, and also showed no difference in routine management between groups, for instance in the use of broad-spectrum antibiotics to treat presumed bacterial infections. Both urine LAM (point-of-care lateral flow assay) and Xpert (approximately 2 h after urine centrifugation) are rapid, as was initiation of tuberculosis treatment in this trial, which are crucial to affect mortality and potentially transmission.

The least costly and easiest urine test (TB-LAM) had major incremental diagnostic benefit in this trial, with urine Xpert (which is more complex because of urine centrifugation) contributing few additional diagnoses. This finding argues for the use of LAM alone as the urinary diagnostic for screening, an approach supported by STAMP's cost-effectiveness projections being reported separately.^25^ Urine Xpert might still have a place alongside other diagnostic modalities for urine LAM-negative patients with high clinical suspicion for tuberculosis.^16^ Sputum Xpert is already a recommended standard of care for HIV-positive individuals with tuberculosis symptoms (although not uniformly implemented), and was included for all patients able to expectorate in both trial groups.^9^ Our data support this approach, because sputum provided the only microbiological diagnosis for 14% of patients with tuberculosis in the intervention group.

Despite the qualified mortality benefits, we consider our results to be supportive of routine implementation of systematic screening with urinary LAM, in addition to sputum Xpert, for all HIV-positive inpatients, given that LAM screening provides a substantial increase in diagnosis of a treatable but frequently fatal condition (disseminated tuberculosis). Systematic screening on admission to hospital is a simple strategy that avoids expense and delay from identifying high-risk groups (including by CD4 count testing, which might not be routinely available).^26^ Tuberculosis symptoms were present in 90% of patients yet only 39% were considered to have tuberculosis by clinicians, who would have missed a substantial number of diagnoses. Notably, 61% of inpatients in STAMP were in one or more high-risk groups with a mortality benefit. Pronounced differences in short-term mortality and underlying prevalence of disseminated tuberculosis between HIV-positive inpatients and outpatients also means that current WHO policy (specific recommendation against use for screening, with use of TB-LAM indicated only for diagnostic purposes in patients with tuberculosis symptoms and CD4 counts <100 cells per μL or signs of severe illness) should remain in use for outpatients.^21^

There are limitations to our study. In sample size calculations, we assumed a higher mortality and burden of tuberculosis than we observed, possibly because of the success of ART scale-up. We did not include a culture reference standard, as this reference is neither standard of care nor routinely available in Malawi, and would have presented ethical dilemmas or affected generalisability. Relatively few participants in Malawi produced sputum. This difficulty in expectorating sputum is, however, typical of unselected HIV-positive outpatient and inpatient cohorts in resource-constrained settings, and is a major barrier to implementation of sputum-based tuberculosis screening.^15^, ^27^ Generalising the true effect of this intervention might be compromised by uncharacteristically prompt specimen collection and results reporting in the study setting, and masking of routine clinicians to exactly which tests had been done might have altered their clinical decision making.^28^ Conversely, because both urine assays were run in a laboratory, we might have underestimated the effect on outcomes from TB-LAM if used at the bedside with faster turnaround times. Patients excluded because of their inability to consent will have introduced bias to the study towards the less critically ill, potentially affecting generalisability.

In conclusion, we report a survival benefit from urine-based tuberculosis screening of HIV-positive hospital admissions in high-risk subgroups, and a broader benefit through substantially increased predischarge tuberculosis diagnosis and treatment in all patients. Tuberculosis screening with urine-LAM lateral flow assays is inexpensive and easily implementable, requiring minimal infrastructure and training. Tuberculosis diagnosed through urine-based screening was complementary to tuberculosis diagnosed through standard clinical investigations in both countries, with inferred higher risk of discharge with undiagnosed tuberculosis in the standard-of-care group than in the intervention group. Anticipated improvements in analytical performance of next-generation LAM assays might add to the diagnostic yield from systematic urine screening.^29^ Current WHO guidelines on the diagnostic use of TB-LAM in HIV-positive inpatients have been insufficient to motivate widespread implementation within African hospitals.^30^ These results support changes to current policy and guidelines for routine inclusion of urine-based tuberculosis screening using TB-LAM in a package of interventions for HIV-positive patients admitted to hospital in high-burden settings, aiming to reduce short-term morbidity and mortality.^31^, ^32^, ^33^ Such new strategies need to be urgently implemented to achieve WHO's End TB Strategy targets of a 75% reduction in tuberculosis mortality by 2025.
