# Supplementary material for: Rapid urine-based screening for tuberculosis in HIV-positive patients admitted to hospital in Africa (STAMP): a pragmatic, multicentre, parallel-group, double-blind, randomised controlled trial
Source: Lancet. 2018 Jul 28;392(10144):292–301. doi: 10.1016/S0140-6736(18)31267-4 (PMC6078909; doi:10.1016/S0140-6736(18)31267-4)
Supplement: Supplementary appendix [file mmc1.pdf]

THE LANCET

Supplementary appendix

This appendix formed part of the original submission and has been peer reviewed. We post it as supplied by the authors.

Supplement to: Gupta-Wright A, Corbett EL, van Oosterhout JJ, et al. Rapid urine-based screening for tuberculosis in HIV-positive patients admitted to hospital in Africa (STAMP): a pragmatic, multicentre, parallel-group, double-blind, randomised controlled trial. *Lancet* 2018; published online June 19. http://dx.doi.org/10.1016/S0140-6736(18)31267-4.

Supplementary web appendix

Rapid urine-based screening for tuberculosis in hospitalised HIV-positive patients in Africa: a pragmatic, parallel-group, blinded randomised controlled trial

Contents

1. **List of investigators** 3
2. **Methods** 4
   - **2.1 Site profile: Zomba Central Hospital, Malawi** 4
   - **2.2 Site profile: Edendale Hospital, KwaZulu-Natal, Africa** 4
   - **2.3 Further details of laboratory methods and TB screening results** 5
   - **2.4 Sample size calculation from the trial protocol** 7
   - **2.5 Trial oversight** 7
   - **2.6 Deviations from the study protocol** 7
3. **Results** 8
   - **Figure 1. A: CONSORT diagram outlining screening, enrolment, randomization and primary endpoint, Edendale, South Africa site** 8
   - **Figure 1. B: CONSORT diagram outlining screening, enrolment, randomization and primary endpoint, Zomba, Malawi site** 9
   - **Table 1A. Additional baseline characteristics, by study arm and by site** 10
   - **Table 1B. Baseline characteristics by study site and stratified by study arm** 11
   - **Figure 2: Primary Outcome including sub-group analysis (Odds Ratios)** 12
   - **Table 2: Number needed to screen** 13
   - **Table 3: Primary outcome and subgroup analysis sensitivity analysis, assuming all patients lost to follow-up died.** 13
   - **Table 4: TB investigations undertaken by study arm and site** 14
   - **Figure 3: Predefined secondary outcomes compared between study arms** 15
   - **Table 5: TB outcomes (during and following hospitalisation) at 56-days by arm** 16
   - **Table 6A: Positive TB diagnostics by study arm** 17
   - **Table 6B: Reasons for starting TB treatment during hospitalisation, by arm and site** 17
   - **Figure 4: TB outcomes (during hospitalisation) and subgroup analyses** 18
   - **Figure 5: Diagnostic yield by study arm** 19
   - **Table 7: Adverse events related to tuberculosis treatment** 20
   - **Table 8: Tuberculosis drug resistance diagnosis** 20
   - **Table 9: Proportion of pre-specified subgroups who were clinically suspected of tuberculosis** 21

1. List of investigators

London School of Hygiene & Tropical Medicine: Stephen Lawn (chief investigator until his death Sept 2016), Katherine Fielding (co-chief investigator), Elizabeth Corbett (co-investigator), Ankur Gupta-Wright (trial coordinator), Clare Flach (trial statistician until August 2016), Daniel Grint (trial statistician from October 2016), Tina Lloyd (project administrator), Seven Kuo (project administrator).

Malawi-Liverpool-Wellcome Trust Clinical Research Program, Blantyre Malawi: Elizabeth Corbett (co-investigator), Ankur Gupta-Wright (trial coordinator), Lingstone Chiume (data manager), George Sinjani (scientific administrator), Elizabeth Chimbayo (study laboratory technician), Doris Shani (laboratory manager), Thandiwe Gondwe (project administrator)

Dignitas International, Zomba Malawi: Joep van Oosterhout (principal investigator), Melanie Alufandika-Moyo (site coordinator), Anthoney Tebulo (study laboratory technician), Deborah Phiri (data coordinator), Timeo Mtenga (clinical officer). Research nurses: Edward Mangani, Madalo Mataka, Dorothy Kazembe and Duncan Kwaitana. Research assistants: Ivy Missi, Boniface Muriya, Wezi Jusu.

Umkhuseli Innovation and Research Management, Pietermaritzburg, South Africa: Doug Wilson (principal investigator), Jurgens Peters (site coordinator until August 2017), Carolin Bresges site coordinator from September 2017), Mduduzi Ngwane (study laboratory technician), Siyabonga Nhlapo (data coordinator), Research nurses: Zanele Macqaba, Zinhle Ncobo, Lihle Mbambo. Research assistants: Londi Musgrave, Donald Xhao.

Trial Steering Committee: Anthony Harries (chair), Andrew Ramsay, Frank Cobelens, Stephen Lawn, Katherine Fielding, Elizabeth Corbett, Joep van Oosterhout, Doug Wilson, Ankur Gupta-Wright, funder representative.

Data Safety Monitoring Board: Andrew Nunn (chair), Anton Pozniak, Tom Harrison

2. Methods

2.1 Site profile: Zomba Central Hospital, Malawi

Location and epidemiology: Zomba is a largely rural district in Southern Malawi with an estimated population of 799,000 in 2015, of whom 138,000 live in Zomba City. HIV prevalence is estimated at 16.8% among 15-49 year olds in 2015. Zomba is one of the highest TB burden districts in Malawi, and notification rates were estimated at 150 per 100,000 in 2015. Over half of the TB notifications occur in Zomba Central Hospital.

Zomba Central hospital: Zomba Central Hospital is one of four central hospitals in Malawi and serves as a district hospital within Zomba district and a tertiary referral centre to 4 other district hospitals. The hospital has 500 beds and 4 main clinical departments: internal medicine, surgery, paediatrics and obstetrics & gynaecology. There is no emergency department, admissions are made through outpatient clinics. The hospital is mostly staffed by clinical officers (having undertaken a 3-year Diploma in Clinical medicine). All routine care is provided free of charge to the user.

Medical Department: The medical department has 160 beds spread over 3 wards (although bed occupancy can be >100%). The inpatient care is delivered by 6 clinical officers, and supervised by 3 medical doctors. The nurse to patient ratio is usually between 1:20 and 1:30. Only basic haematology (full blood count, cross-matching, CD4 count), biochemistry (renal and liver function), microbiology (microscopy), serology (hepatitis and syphilis) and HIV-1 viral load testing are available, although there are frequent disruptions when these services are not available. The radiology department offers radiography and ultrasound during office working hours. Complex patients can be referred to the national referral centre (Queen Elizabeth Central Hospital) in Blantyre (approximately 60km away).

TB diagnostics: Routine TB diagnostics available on-site include sputum smear microscopy and Xpert MTB/RIF assays (during office working hours only). Only respiratory samples are processed for Xpert, and no mycobacterial culture facilities are available. Patients with suspected MDR-TB (eg Xpert rifampicin resistance samples) are managed by the National TB Programme, and samples are sent to Lilongwe for culture and first line drug sensitivity testing (second line drugs are centralized at national level in Malawi). Sputum induction is not available. CSF can be processed for microscopy, cryptococcal antigen testing and India ink staining only. No other routine TB diagnostics are available.

HIV care: Malawi has implemented universal 'test and treat' since mid-2016, prior to that the threshold for ART was a CD4 cell count of 500 cells/µL. HIV care is usually delivered through local health centres. Monitoring is monthly for patients newly initiated for 6 months, then 2 monthly for 6 months, and thereafter 3 monthly. Viral load monitoring is recommended but CD4 cell count monitoring is not available. The first line ART regimen is tenofovir, lamivudine and efavirenz as a fixed-dose combination.

TB and HIV/TB care: TB is treated using the standard WHO recommended 4-drug regimen for 6 months (a 2-month intensive phase with isoniazid, rifampicin, pyrazinamide, and ethambutol, followed by a 4-month continuation phase with isoniazid and rifampicin). Patients being treated for a second time, or failing their first course of treatment are managed with streptomycin added to the intensive phase, usually requiring hospitalisation and daily intramuscular injections for 60-days. HIV/TB is managed by TB clinics, except for at Zomba Central Hospital where there is an integrated HIV/TB clinic. ART is commenced within 2 weeks if ART naïve using the first line regimens. After discharge, HIV/TB patients are seen monthly at Zomba Central Hospital for clinical review and drug refills, unless they live nearer a peripheral health facility in which case they will be referred there for HIV/TB care.

Hospital catchment area: Patients were included in the hospital catchment area if they resided in Zomba district.

2.2 Site profile: Edendale Hospital, KwaZulu-Natal, Africa

Location and epidemiology: Edendale Hospital is a regional level public-sector institution, situated in the peri-urban outskirts of the uMgungundlovu District in Pietermaritzburg, KwaZulu-Natal, South Africa. About 1 million individuals are served by the hospital through a network of eighteen primary healthcare clinics and four district hospitals. There is a high burden of HIV and TB- the uMgungundlovu District has a TB notification rate of 678 cases per 100,000 population and antenatal HIV prevalence of 44%.

Edendale hospital: With a 900 bed capacity, Edendale is South Africa's 4th largest hospital. A level 2 healthcare centre, Edendale provides specialist support to Level 1 facilities. These include 4 referring district hospitals, 2 TB hospitals, and an additional 18 Primary and Community Health Care Centres. Specialist services include emergency medicine, general internal medicine, paediatrics, general and specialist surgery, obstetrics and gynaecology, ophthalmology and psychiatry. Services are charged for on an income-based sliding scale and are essentially free to patients who are unable to pay.

Medical Department: There are 234 inpatient beds across 7 wards. Admissions are from the emergency department, ambulatory care or outpatient department and referrals from other hospitals. Internal medicine is staffed by 40 doctors, including eight specialist physicians, fourteen medical officers, and eighteen medical interns. Intern doctors are responsible for approximately 15-20 patients. Nursing care is provided by approximately 140 nurses, at an approximate nurse:patient ratio of 1:8. Inpatient HIV prevalence is estimated to be 35-50%, with TB incidence estimated to be 15%. Laboratory testing includes full biochemistry, haematology and microbiology services. Inpatient point of care HIV testing is performed by ward staff.

TB diagnostics: Routine TB diagnostics at Edendale include onsite fluorescence microscopy for acid fast bacilli and Xpert MTB/RIF (respiratory and non-respiratory samples) during working hours, with results usually available within 48 hours. Mycobacterial culture is available (off-site) for respiratory and non-respiratory samples, and drug-resistance testing for suspected drug resistant TB (eg Xpert rifampicin resistance samples). Sputum induction is not routinely done, but is available if requested. Cytology and histology are available. Biochemical and cell analysis is performed on extra-pulmonary specimens, including cerebrospinal, pleural and ascitic fluid. Cytology and histology services are also available. Chest radiography is available 24 hours per day. Other radiology services include ultrasound and CT scanning, available during working hours.

HIV care: In January 2015, the ART treatment threshold was 500 cells/μL, and from September 2016 universal test and treatment was introduced, prioritising patients with CD4 cell counts <350 cells/μL. First line ART currently consists of Tenofovir, Emtricitabine and Efavirenz as a fixed-dose combination pill. H HIV care is managed at the primary healthcare clinics and is primarily nurse led with doctor input. Laboratory testing includes CD4 cell count and viral load monitoring as per South African national guidelines.

TB and HIV/TB care: TB is treated using the standard rifamycin based 4-drug regimen for 6 months (a 2-month intensive phase with isoniazid, rifampicin, pyrazinamide, and ethambutol, followed by a 4-month continuation phase with isoniazid and rifampicin). Continuation phase is extended to 7 months for TB meningitis. HIV/TB care is managed by the primary healthcare clinics, including provision of ART. Treatment naïve patients are usually prescribed ART within 2-8 weeks of TB diagnosis, often during hospital admission, with cotrimoxazole prophylaxis.

Hospital catchment area: Patients were included in the hospital catchment area if they resided in uMgungundlovu District.

2.3 Further details of laboratory methods and TB screening results

HIV-testing: HIV testing was done according to local protocols and guidelines, and used rapid, point-of-care tests with a second test for confirmation. Zomba, Malawi, used the Determine HIV 1/2 and Unigold HIV Rapid Test in a sequential algorithm. Edendale, South Africa used the Advanced Quality Rapid Anti-HIV (1&2) Test followed by confirmatory testing using the Reveal G2 Rapid HIV-1 Antibody Test.

Determine TB-LAM methodology: LAM strip tests were stored securely in the research laboratory according the manufacturer's recommendations (between 4 and 25°C). Each batch was tested with serially diluted LAM antigen solution to check performance prior to use. Laboratory technicians were trained in performing and reading LAM strips by the international trial coordinator, who regularly monitored proficiency.

TB-LAM assay was performed as per the manufacturer's instructions. Urine arrived in the laboratory in a 50ml container, and 60μL of neat/unprocessed urine was pipetted onto the sample pad of new TB-LAM strip. A timer was set for 25-minutes, after which the validity of the test was assessed by checking for a band in the control window. The test result was then assessed by comparing the intensity of the band in the sample window (if present) with the manufacturer's reference card. If the band was as or more intense than the lightest positive band (grade 1 on the post 2014 reference card), the test was deemed positive. The positive results were graded (grade 1-4) according to the band which the sample window was as intense as (see figure). Non-valid tests were repeated. TB-LAM test strips were stored and a random sample independently double-read (this was done

masked to the original result) for quality assurance. TB-LAM tests were read prior to the results of the Xpert MTB/RIF and without knowing any clinical details of the patient to reduce bias.

Urine Xpert MTB/LAM methodology: Following the removal of 60 µL urine for TB-LAM testing and up to 7mls for storage, the remaining urine (usually 40-50mls) was centrifuged at 3000g for 15mins in a bucket centrifuge. The supernatant was discarded, and the urine pellet resuspended in approximately 2ml of residual urine/supernatant. 0.75ml of the resuspended pellet was added to 1.25ml of Xpert MTB/RIF sample reagent and incubated for 15 minutes, then added to the cartridge and processed as per the manufacturer's instructions.

Sputum Xpert MTB/RIF assay was performed as per the manufacturer's instructions.

Issuing of results: once all the study samples had been processed for TB assays according to study arm allocation, the study laboratory technician reported the results on a study results sticker as 'STAMP TB screening positive', 'STAMP TB screening negative' or 'STAMP TB screening not done'. A positive result was reported if any assay (TB-LAM or Xpert) was positive. Xpert rifampicin resistance results were also reported as 'Rifampicin resistance mutations not detected', 'Rifampicin resistance mutations DETECTED', or 'Rifampicin not available'. These results stickers were placed in the routine medical notes by study nurses (all remained masked to study arm), and if members of the routine medical team were available on the ward they were notified of the results by the study nurses. The individual test results or study arm were never communicated to the attending clinicians or the study teams.

The routine medical staff received training on how to interpret the STAMP TB results, including the assays done in each arm, diagnostic accuracy and expected positive and negative predictive value. Training was repeated for new staff and periodically during the study period.

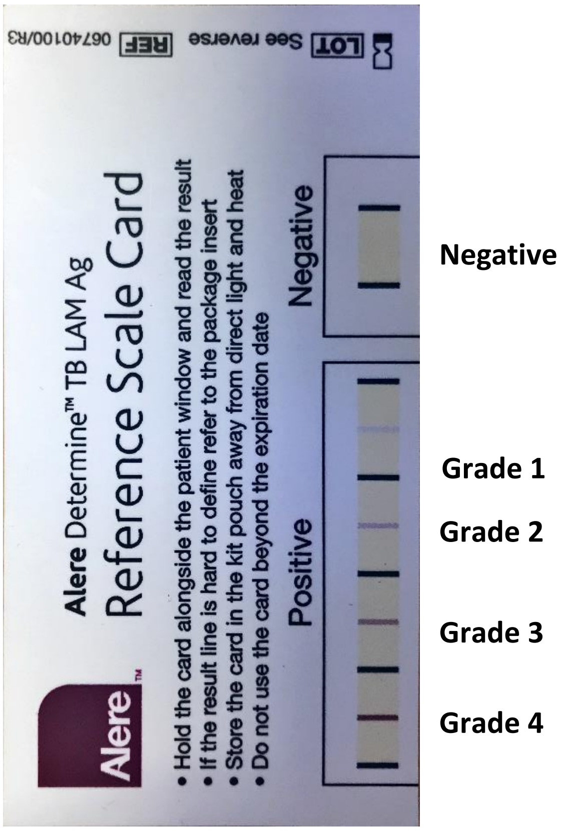

2.4 Sample size calculation from the trial protocol

The sample size calculation is based on the primary endpoint of mortality risk by 56 days from randomisation. Assuming an all-cause mortality of 25% in the control arm after 56 days of follow-up (based on unpublished data collected from both sites) and a 2-sided type I error of 5%, inclusion of 1,300 patients per arm would provide 90% power to detect a 25% reduction in mortality and 80% power to detect a 20% reduction, allowing for loss to follow-up (LTFU) of 10%-15% by 56 days (see table 11.1). If the 56 day mortality risk in the control arm were unexpectedly lower (eg 20%), then this sample size would still be sufficient to provide 80% power to detect a 25% reduction in all-cause mortality with 15% LTFU. The study will have greater power for the secondary endpoint of time to death, measured up to 56 days from randomisation. The power calculation was based on the mortality risk for the entire cohort and was not powered to detect mortality differences between sites or pre-specified clinical subgroups.

**All-cause mortality SOC arm**	**All-cause mortality: Intervention arm**	**Number of patients per arm**			
		**Power 80% 10% LTFU**	**Power 80% 15% LTFU**	**Power 90% 10% LTFU**	**Power 90% 15% LTFU**
20%	16.0%	1663	1761	2208	2338
20%	15.0%	1050	1112	1391	1473
20%	14.0%	719	761	950	1006
25%	20.0%	1260	1334	1671	1769
25%	18.75%	798	845	1056	1118
25%	17.5%	548	580	722	765
30%	24.0%	991	1049	1313	1391
30%	22.5%	629	666	831	880
30%	21.0%	432	458	570	604

Table. Sample-size calculations

2.5 Trial oversight

London School of Hygiene & Tropical Medicine was the trial sponsor. Independent trial steering committee and data and safety monitoring board members reviewed trial progress, and for the latter, interim outcome data 6-monthly. The trial was prospectively registered (ISRCTN71603869).

The trial protocol was approved by the London School of Hygiene & Tropical Medicine Research Ethics Committee (reference 9360), The University of Malawi College of Medicine Research Ethics Committee (reference P.06/15/1743) and the University of KwaZulu-Natal Biomedical Research Ethics Committee (reference BFC215/15).

2.6 Deviations from the study protocol

During the trial, 25 patients were inadvertently enrolled and randomised for a second time during a readmission to hospital, and one patient was randomised and subsequently discovered to be HIV-negative. These 26 patients were excluded from the analysis. Randomisation occurred out of order twice due to accidently skipped randomisation envelopes, and one patient was enrolled and subsequently found to have taken IPT in the preceding 6 months. There were no other deviations from the study protocol.

3. Results

Figure 1. A: CONSORT diagram outlining screening, enrolment, randomization and primary endpoint, Edendale, South Africa site

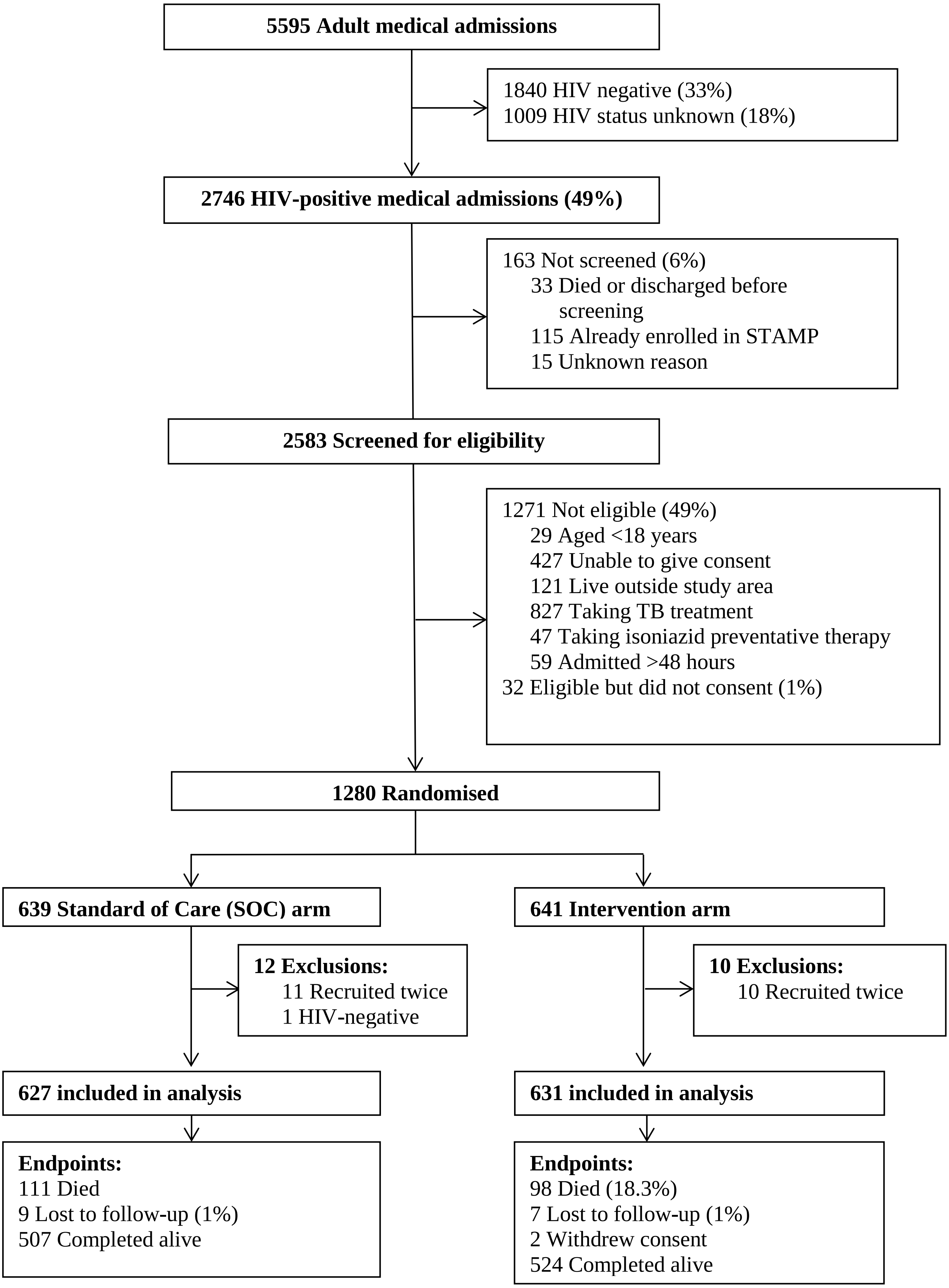

Patients could have more than one reason for exclusion.

Figure 1. B: CONSORT diagram outlining screening, enrolment, randomization and primary endpoint, Zomba, Malawi site

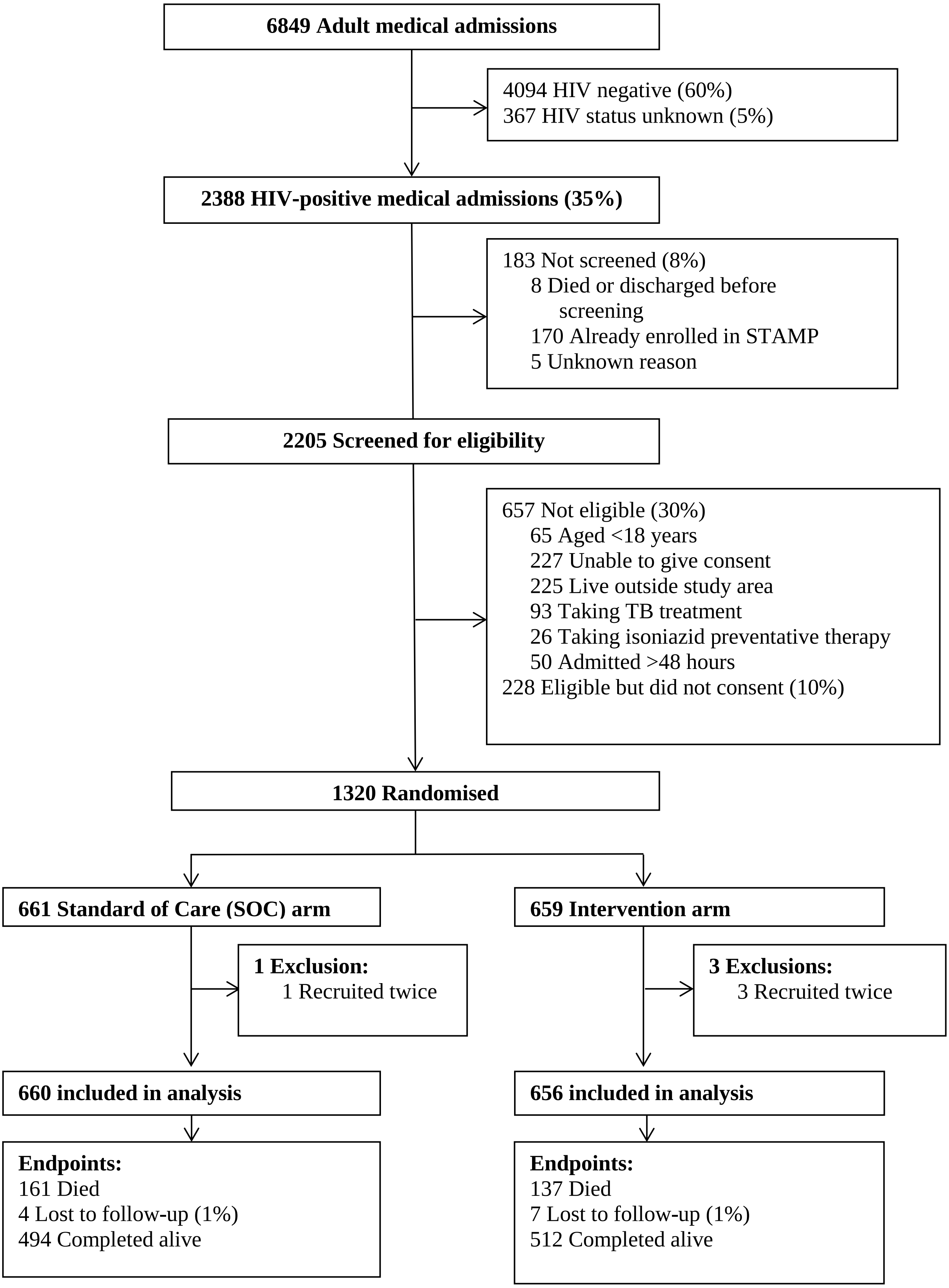

Patients could have more than one reason for exclusion.

Table 1A. Additional baseline characteristics, by study arm and by site

Characteristic	SOC (N=1287)	Intervention (N=1287)	Malawi (N=1316)	South Africa (N=1258)
Smoking Status				
Current smoker	128 (9.9)	151 (11.7)	67 (5.1)	212 (16.9)
Former smoker	220 (17.1)	208 (16.2)	141 (10.7)	287 (22.8)
Never smoked	939 (73.0)	928 (72.1)	1108 (84.2)	759 (60.3)
Cough	681 (52.9)	651 (50.6)	611 (46.4)	721 (57.3)
Producing Sputum	467/689 (67.8)	447/665 (67.2)	370/624 (59.3)	544/730 (74.5)
Coughing Blood	88/685 (12.8)	89/660 (13.5)	48/620 (7.7)	129/725 (17.8)
Taking co-trimoxazole therapy	552/1064 (51.9)	532/1084 (49.1)	954/1104 (86.4)	130/1044 (12.5)
Hepatitis, cirrhosis or liver disease	37 (2.9)	35 (2.7)	31 (2.4)	41 (3.3)
Kidney failure or damage	70 (5.4)	60 (4.7)	9 (0.7)	121 (9.6)
Diabetes mellitus	53 (4.1)	58 (4.5)	17 (1.3)	94 (7.5)
High blood pressure or heart disease	180 (14.0)	182 (14.1)	139 (10.6)	223 (17.7)
Time HIV Diagnosed- Mean (Years)- Mean (SD)	N=1061; 4.9 (4.5)	N=1080; 5.0 (5.4)	N=1094; 4.7 (4.1)	N=1047; 5.2 (5.6)
Weight (kg - Mean (SD)	56.9 (15.4)	56.7 (15.6)	51.0 (10.8)	62.9 (17.2)
Height (cm)- Mean (SD)	161.8 (8.8)	162.1 (8.4)	159.5 (7.9)	164.5 (8.5)
Mid-upper arm circumference (cm) - Mean (SD)	23.3 (5.2)	23.1 (4.7)	23.3 (4.4)	23.1 (5.4)
Heart rate- Mean (SD)	94.0 (19.2)	N=1285; 94.7 (19.4)	N=1314; 92.6 (18.4)	96.1 (20.0)
Systolic blood pressure (mmHg)- Mean (SD)	111.7 (20.4)	111.0 (20.8)	107.5 (20.3)	115.4 (20.2)
Diastolic blood pressure (mmHg)- Mean (SD)	72.5 (14.7)	71.3 (14.6)	71.6 (14.8)	72.3 (14.4)
Respiratory rate (breaths per minute)- Mean (SD)	N=1285; 22.2 (4.8)	N=1284; 22.2 (4.5)	N=1315; 22.5 (5.2)	N=1254; 21.8 (3.9)
Oxygen saturation (%)- Mean (SD)	96.3 (25.5)	N=1284; 95.7 (4.2)	N=1315; 96.4 (25.2)	N=1256; 95.7 (4.7)
On supplementary oxygen	71 (5.5)	75 (5.8)	6 (0.5)	140 (11.1)
Temperature (C) - Mean (SD)	36.4 (0.7)	36.5 (0.7)	36.4 (0.8)	36.5 (0.6)

Numbers are n (%) unless otherwise stated. Denominator is given where data is missing. SD indicates standard deviation, SOC standard of care.

Table 1B. Baseline characteristics by study site and stratified by study arm

	Zomba (N=1316)		Edendale (N=1258)	
Characteristic	SOC (N=660)	Intervention (N= 656)	SOC (N=627)	Intervention (N= 631)
Age (years), mean (SD)	40.2 (11.8)	40.1 (11.7)	39.0 (12.0)	39.3 (11.5)
Gender				
Female	419 (63.5)	410 (62.5)	315 (50.2)	317 (50.2)
New HIV diagnosis	105 (15.9)	103 (15.7)	107 (17.1)	91 (14.4)
ART status[a]				
Never	23 (4.1)	34 (6.1)	70 (13.5)	87 (16.1)
Currently taking	515 (92.8)	506 (91.5)	420 (80.8)	420 (77.8)
Interrupted	17 (3.1)	13 (2.4)	30 (5.8)	33 (6.1)
Time on ART (years), median (IQR)[b]	3.5 (0.8-7.4)	3.3 (0.8-7.5)	2.3 (0.4-5.8)	2.7 (0.8-5.8)
TB symptoms reported				
Cough	324 (49.1)	287 (43.8)	357 (56.9)	364 (57.7)
Fever	385 (58.3)	376 (57.3)	362 (57.7)	377 (59.7)
Night sweats[c]	269 (40.8)	219 (33.4)	271 (43.3)	278 (44.1)
Weight loss[c]	425 (64.4)	438 (66.8)	450 (71.9)	468 (74.2)
Any WHO TB symptom	599 (90.8)	588 (89.6)	565 (90.1)	564 (89.4)
TB clinically suspected[d]	184 (28.0)	169 (26.0)	311 (49.6)	332 (52.6)
Previous TB treatment	103 (15.6)	99 (15.1)	206 (32.9)	236 (37.4)
Body Mass Index, mean (SD)	20.1 (4.1)	19.9 (4.1)	23.4 (6.8)	23.3 (6.7)
Morbidity at admission				
WHO danger sign[e]	163 (24.7)	174 (26.5)	112 (17.9)	103 (16.3)
Karnofsky score, median (IQR)	60 (50-70)	60 (50-70)	60 (50-70)	60 (50-70)
CD4 cell count (cells/µL) [d]				
median (IQR)	222 (93-436)	218 (79-428)	223 (68-436)	247 (72-461)
< 100	178 (27.1)	187 (28.5)	199 (32.0)	184 (29.2)
Haemoglobin (g/dL) [f]				
median (IQR)	105 (77-125)	102 (79-124)	113 (88-132)	112 (87-130)
< 8	186 (28.2)	169 (25.8)	112 (17.9)	120 (19.1)

Numbers are n (%) unless otherwise stated. [a] ART status denominator is patients with a known HIV diagnosis. [b] Missing data for 26 patients in Malawi and 26 in South Africa. [c] Missing data for 1 patient in South Africa. [d] Missing data for 3 patients in Malawi and 6 in South Africa. [e] WHO danger signs are ≥1 of: heart rate>120 beats per minute, respiratory rate>30 breaths per minute, temperature over 39°C or being unable to walk unaided. [f] Missing data for 5 patients in South Africa. TB symptoms are reported if present for any duration. ART indicates antiretroviral therapy, HIV human immunodeficiency virus, IQR interquartile range, SD standard deviation, SOC standard of care, WHO World Health Organization.

Figure 2: Primary Outcome including sub-group analysis (Odds Ratios)

Primary Outcome	56-Day Mortality SOC n (%)	56-Day Mortality Intervention n (%)	aOR (95% CI)	P-Value	Interaction P-Value
Overall mortality	272 (21.1)	235 (18.3)	0.83 (0.69, 1.01)	0.0679	
Subgroup analyses:					
Site					0.8279
Malawi	161 (24.4)	137 (20.9)	0.82 (0.63, 1.06)	0.1285	
South Africa	111 (17.7)	98 (15.5)	0.85 (0.63, 1.15)	0.3009	
Calendar time					0.5003
Oct 2015 - Mar 2016	59 (21.8)	53 (19.8)	0.89 (0.58, 1.34)	0.5689	
Apr 2016 - Sep 2016	75 (20.9)	75 (21.1)	1.01 (0.70, 1.45)	0.9559	
Oct 2016 - Mar 2017	74 (20.8)	60 (16.9)	0.78 (0.53, 1.13)	0.1864	
Apr 2017 - Sep 2017	64 (21.1)	47 (15.2)	0.67 (0.44, 1.01)	0.0600	
CD4 cell count, cells/µL					0.1628
<100	133 (35.7)	107 (28.8)	0.72 (0.53, 0.98)	0.0386	
≥100	131 (14.6)	127 (14.0)	0.96 (0.74, 1.25)	0.7766	
Haemoglobin, g/dL					0.1222
<8	116 (38.9)	86 (29.8)	0.67 (0.47, 0.94)	0.0221	
≥8	156 (15.8)	149 (15.0)	0.94 (0.73, 1.19)	0.5909	
TB suspected at admission					0.1894
Clinically suspected TB	136 (27.5)	106 (21.2)	0.72 (0.54, 0.97)	0.0286	
TB not suspected	136 (17.2)	128 (16.4)	0.94 (0.72, 1.22)	0.6381	

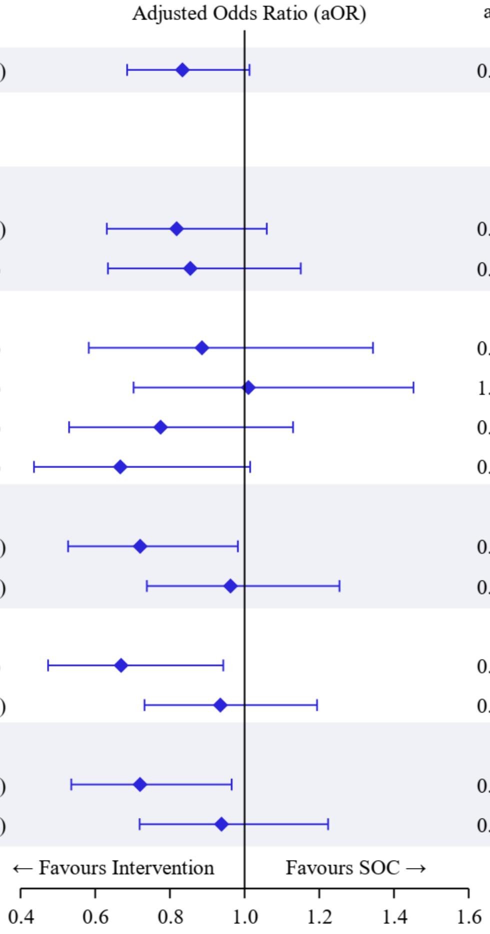

Primary outcome is mortality at 56-days post randomisation. Odds ratios are odds in intervention compared to SOC arms. n is the number of patients with outcome. All analyses are adjusted only for study site. SOC indicates Standard of Care, aOR adjusted odds ratio.

Table 2: Number needed to screen

Group Screened	NNTS to prevent 1 death	NNTS to prevent 1 missed TB diagnosis
All patients	35.7	13.7
Baseline CD4 count <100 cells/µL	14.1	12.7
Severe anaemia at admission	11.1	5.4
Clinically suspected of TB	17.5	21.3
All patients, Malawi site only	28.6	11.2

NNTS indicates number needed to screen with the study intervention.

Table 3: Primary outcome and subgroup analysis sensitivity analysis, assuming all patients lost to follow-up died.

	N	Risk difference* % (95% CI)	P-Value	Interaction P-Value
**Intervention - Control**	2574	-2.75 (-5.86, 0.36)	0.0827	
**Subgroup analyses:**				
**Site**				0.8627
**Zomba**	1316	-3.05 (-7.63, 1.53)	0.1917	
**Edendale**	1258	-2.50 (-6.74, 1.74)	0.2474	
**Calendar time**				0.6972
**OCT15 - MAR16**	539	-2.34 (-9.30, 4.62)	0.5084	
**APR16 - SEP16**	714	-0.29 (-6.25, 5.71)	0.9242	
**OCT16 - MAR17**	709	-4.29 (-10.19, 1.61)	0.1532	
**APR17 - SEP17**	612	-4.26 (-10.34, 1.72)	0.1645	
**Baseline CD4 cell count**				0.0681
**CD4 < 100**	744	-7.13 (-13.79, -0.42)	0.0367	
**CD4 ≥ 100**	1801	-0.12 (-3.44, 3.19)	0.9455	
**Baseline haemoglobin**				0.0706
**Haemoglobin < 8.0g/dL**	587	-8.62 (-16.27, -0.90)	0.0281	
**Haemoglobin ≥ 8.0g/dL**	1982	-0.93 (-4.18, 2.33)	0.5769	
**TB suspected at admission**				0.1209
**Clinically suspected TB**	996	-5.74 (-11.10, -0.39)	0.0356	
**TB not suspected**	1569	-0.76 (-4.48, 2.97)	0.6885	

Primary outcome is mortality at 56-days post randomisation. Risk differences are intervention minus SOC arms. All analyses are adjusted for study site. SOC indicates Standard of Care, CI confidence interval. Interaction p-values are for interaction between study arm and subgroup.

Table 4: TB investigations undertaken by study arm and site

TB Investigations	SOC (N=1287) n (%)	Intervention (N=1287) n (%)	Malawi (N=1316) n (%)	South Africa (N=1258) n (%)
Study samples (taken at enrolment):				
Sputum Xpert	727 (56.5)	722 (56.1)	517 (39.3)	932 (74.1)
Urine Xpert	0	1275 (99.1)	651 (49.5)	624 (49.6)
Urine TB-LAM	0	1275 (99.1)	651 (49.5)	624 (49.6)
Routine TB investigations (during course of inpatient stay):				
CXR	613 (47.6)	618 (48.0)	300 (22.8)	931 (74.0)
Non-study sputum Xpert	184 (14.3)	210 (16.3)	61 (4.6)	333 (26.5)
Non-study other Xpert	11 (0.9)	13 (1.0)	1 (0.1)	23 (1.8)
Sputum culture	134 (10.4)	145 (11.3)	0	279 (22.2)
Other culture	74 (5.7)	77 (6.0)	1 (0.1)	150 (11.9)
CSF	178 (13.8)	176 (13.7)	199 (15.1)	155 (12.3)
Lymph node aspirate	5 (0.4)	5 (0.4)	5 (0.4)	5 (0.4)
Sputum smear	184 (14.3)	210 (16.3)	61 (4.6)	333 (26.5)
Other smear	34 (2.6)	34 (2.6)	4 (0.3)	64 (5.1)
Pleural tap	28 (2.2)	21 (1.6)	8 (0.6)	41 (3.3)

Routine investigations were arranged by the routine medical team, independent of the study team. SOC indicates standard of care, CXR chest x-ray, LAM Determine TB-LAM assay, Xpert is Xpert MTB/RIF assay, CSF cerebral spinal fluid.

Figure 3: Predefined secondary outcomes compared between study arms

A. Secondary outcomes reported as time to event

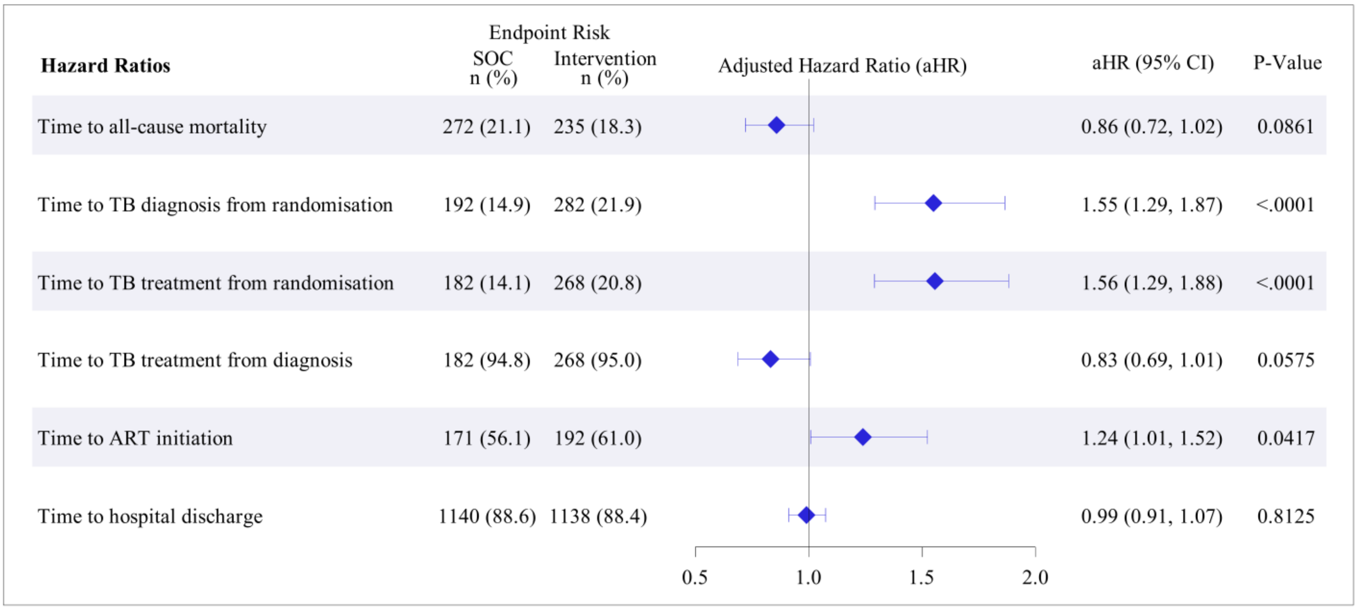

B. Secondary outcomes reported as risk at 56-days

	N		Cumulative Incidence (95% CI)		P-value[a]
	**SOC**	**Intervention**	**SOC**	**Intervention**	
Duration of hospital stay-median days (IQR)	6 (2-11)	6 (2-12)			
Hospital readmission	144	132	13.3% (11.3-15.4)	11.9% (10.1-13.9)	0.48
Losses to follow-up	13	14	1.0% (0.6-1.7)	1.1% (0.6-1.8)	0.84

Values are numbers or proportion unless otherwise stated. TB outcomes were during hospital admission. Mortality, ART initiation, hospital readmission and losses to follow-up were measured at 56-days. All analyses are adjusted for study site. [a] P-value comes from Gray's test comparing cumulative incidence functions, where death has been accounted for as a competing risk. SOC indicates standard of care, aHR adjusted hazard ratio, CI confidence interval, LTFU losses to follow-up, IQR interquartile range.

Table 5: TB outcomes (during and following hospitalisation) at 56-days by arm

Endpoint<br>Intervention - Control	Endpoint Risk<br>SOC<br>n (%)	 Intervention<br>n (%)	Risk Difference[a]<br>% (95% CI)	P-Value
TB Diagnosis (56-day follow-up)	208 (16.2)	293 (22.8)	6.9 (3.9, 9.9)	<0.0001
Microbiologically confirmed TB (56-day follow-up)[b]	92 (7.1)	215 (16.7)	9.8 (7.4, 12.3)	<0.0001
Clinically diagnosed TB (56-day follow-up)[b]	114 (8.9)	77 (6.0)	-3.2 (-5.1, -1.3)	0.0008
Treated for TB (56-day follow-up)	203 (15.8)	288 (22.4)	6.9 (3.9, 9.9)	<0.0001
TB Diagnosed post-discharge[c]	16 (1.2)	11 (0.9)	-0.5 (-1.4, 0.4)	0.31

TB outcomes reported include those during inpatient stay and after discharge up to 56-days post from randomisation, except for TB diagnoses post-discharge. All are exploratory analyses. [a] All models adjusted for study site. [b] Missing data on basis of TB diagnosis for 3 patients who were diagnosed with TB and started on TB treatment post-discharge. [c] The denominator for TB diagnoses post-discharge is patients discharged alive from hospital without a TB diagnosis. SOC indicates standard of care.

Table 6A: Positive TB diagnostics by study arm

	SOC (N=1287)	Intervention (N=1287)	Malawi (N=1316)	South Africa (N=1258)
**TB diagnosis**	**N (%)**	**N (%)**	**N (%)**	**N (%)**
**Diagnosed with TB as in-patient**	192 (14.9)	282 (21.9)	194 (14.7)	280 (22.3)
**Microbiologically confirmed**	85 (44.3)	210 (74.5)	144 (74.2)	151 (53.9)
Sputum Xpert positive*	82 (96.5)	85 (40.5)	59 (41.0)	108 (71.5)
STAMP Xpert positive	68 (82.9)	71 (83.5)	42 (71.2)	97 (89.8)
Non-study Xpert positive	33 (40.2)	23 (27.1)	28 (47.5)	28 (25.9)
Urine TB-LAM positive	NA	158 (75.2)	94 (65.3)	64 (42.4)
Urine Xpert positive	NA	74 (35.2)	37 (25.7)	37 (24.5)
Culture positive	1 (1.2)	0	0	1 (0.7)
Other microbiological test	2 (2.4)	1 (0.5)	1 (0.7)	2 (1.3)

Other microbiological tests include smear microscopy for acid fast bacilli and Xpert on non-sputum and non-urine samples. A TB diagnosis can be positive on more than one test. *Any sputum Xpert positive, including STAMP trial tests or non-study (routine) hospital Xpert tests, some patients were tested with both study and non-study sputum Xpert. Denominator for % is the number of patients one level above. SOC indicates standard of care, TB-LAM Determine TB-LAM assay, Xpert denotes Xpert MTB/RIF assay, NA not applicable (as urine diagnostics were not performed on SOC arm patients).

Table 6B: Reasons for starting TB treatment during hospitalisation, by arm and site

	TB treatment initiations (N=450)			
**Main basis for starting TB Rx**	**SOC (N=182)**	**Intervention (N=268)**	**Zomba (N=185)**	**Edendale (N=265)**
Study result	58 (31.9)	187 (69.8)	122 (65.9)	123 (46.4)
Sputum TB culture	1 (0.5)	0	0	1 (0.4)
Sputum Xpert test (non-study)	13 (7.1)	7 (2.6)	13 (7.0)	7 (2.6)
Other Xpert test (non-study)	1 (0.5)	0	0	1 (0.4)
Other microbiology test	1 (0.5)	0	1 (0.5)	0
Chest x-ray	59 (32.4)	30 (11.2)	34 (18.4)	55 (20.8)
Lumbar puncture/CSF	8 (4.4)	7 (2.6)	1 (0.5)	14 (5.3)
Clinical findings	17 (9.3)	14 (5.2)	10 (5.4)	21 (7.9)
Other	24 (13.2)	23 (8.6)	4 (2.2)	43 (16.2)

Numbers are all N (%). SOC indicates standard of care, CXR chest x-ray, LAM Determine TB-LAM assay, Xpert is Xpert MTB/RIF assay, CSF cerebral spinal fluid.

Figure 4: TB outcomes (during hospitalisation) and subgroup analyses

TB Outcomes	SOC n (%)	Intervention n (%)	aRD (95% CI)	P-Value	Interaction P-Value
TB diagnosis	192 (14.9)	282 (21.9)	7.3 (4.4, 10.2)	<0.0001	
TB treatment	182 (14.1)	268 (20.8)	7.0 (4.1, 9.8)	<0.0001	
TB diagnosis subgroup analyses:					
Site					0.1927
Malawi	68 (10.3)	126 (19.2)	8.9 (5.1, 12.7)	<0.0001	
South Africa	124 (19.8)	156 (24.7)	4.9 (0.4, 9.5)	0.0346	
CD4 cell count, cells/µL					0.8759
<100	116 (31.1)	142 (38.3)	7.9 (1.1, 14.5)	0.0213	
≥100	74 (8.2)	137 (15.2)	7.0 (4.1, 10.0)	<0.0001	
Haemoglobin, g/dL					0.0002
<8	58 (19.5)	111 (38.4)	18.6 (11.5, 25.6)	<0.0001	
≥8	133 (13.5)	171 (17.2)	4.1 (1.0, 7.2)	0.0098	
TB suspected at admission					0.3233
Clinically suspected TB	143 (28.9)	170 (33.9)	4.7 (-1.1, 10.4)	0.1099	
TB not suspected	49 (6.2)	111 (14.2)	8.0 (5.0, 11.1)	<0.0001	

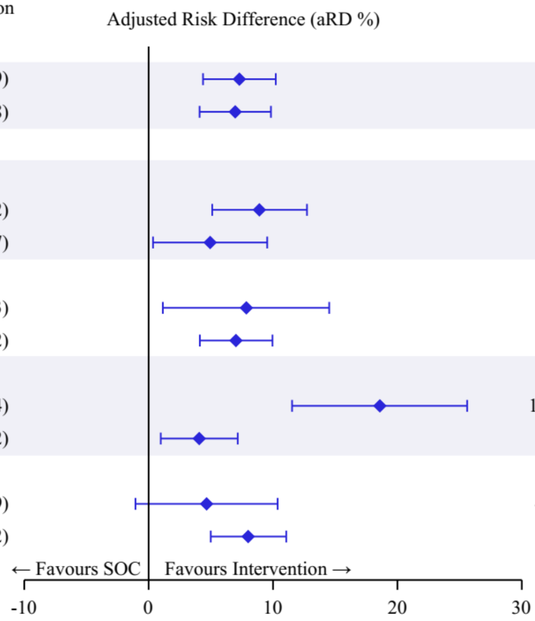

Exploratory analysis of TB diagnosis by subgroup. Risk differences are intervention minus SOC arms. n is the number of patients with TB diagnosis. TB outcomes are measured at the end of hospital admission. All analyses are adjusted only for study site. TB outcomes at 56-days are reported in Table 5, Appendix. SOC indicates Standard of Care, aRD adjusted risk difference, CI confidence interval.

Figure 5: Diagnostic yield by study arm

A. Diagnostic yield for all microbiologically confirmed TB in each arm

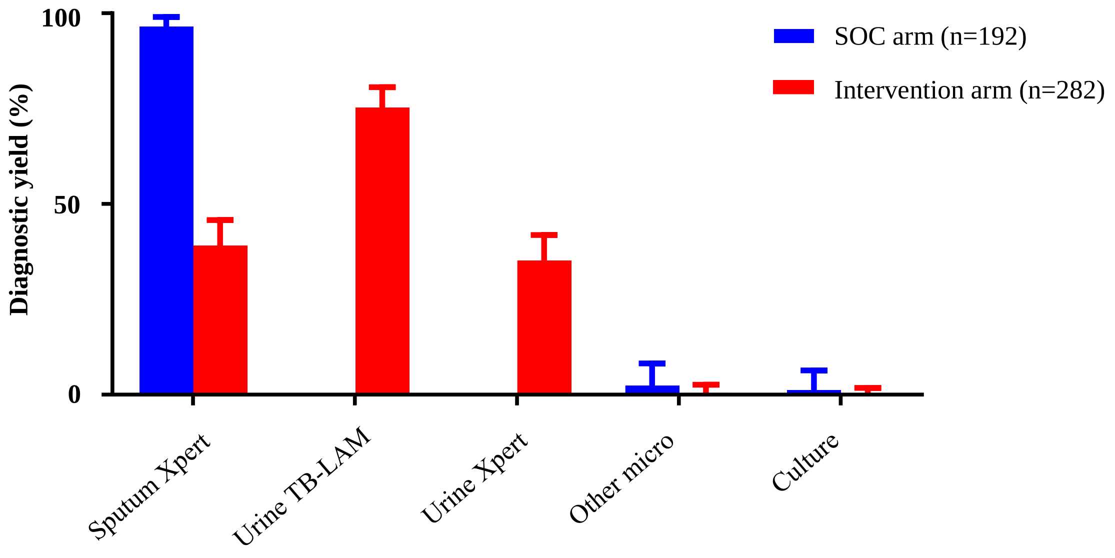

Graph shows the proportion (%) of microbiologically-confirmed TB diagnoses which were diagnosed by each test. There were 85 microbiologically confirmed TB diagnoses in the SOC arm and 210 in the intervention arm. Error bars represent 95% confidence intervals. A TB diagnosis can be positive on more than one test. Other micro includes smear microscopy for acid fast bacilli and Xpert on non-sputum and non-urine samples. SOC indicates Standard of Care, TB-LAM is Determine TB-LAM assay, Xpert is Xpert MTB/RIF assay.

B. Diagnostic yield for all in-patient TB diagnoses in each arm

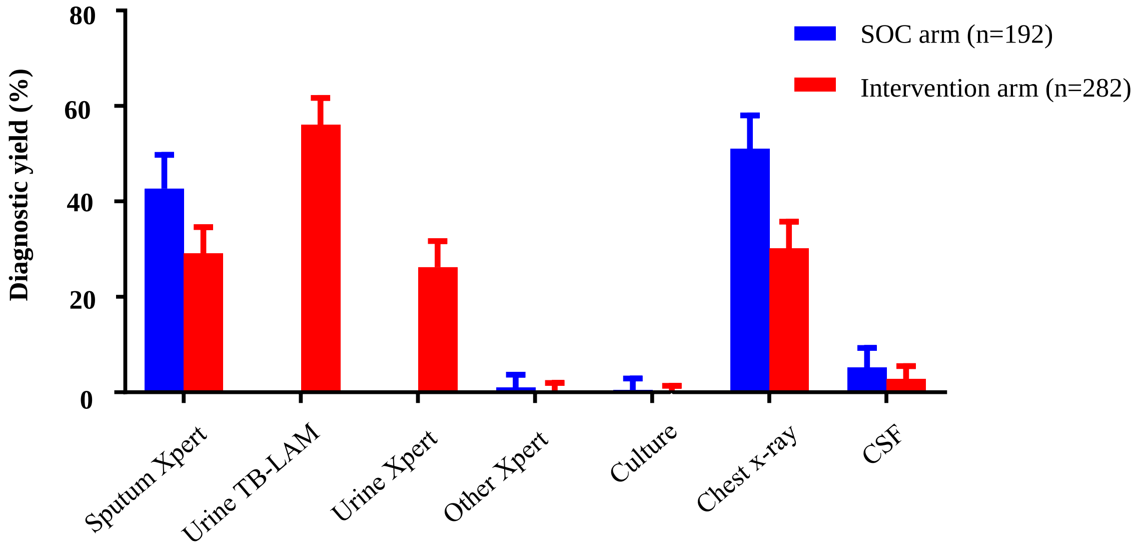

Graph shows the proportion (%) of all inpatient TB diagnoses which were diagnosed by each test. Error bars represent 95% confidence intervals. There were 192 TB diagnoses in the SOC arm and 282 in the intervention arm. CXR is positive if reported by clinicians as consistent with TB. CSF is positive if cited as a reason for starting TB treatment. A TB diagnosis can be positive on more than one test. SOC indicates Standard of Care, CXR chest x-ray, TB-LAM Determine TB-LAM assay, Xpert Xpert MTB/RIF assay, CSF cerebral spinal fluid.

Table 7: Adverse events related to tuberculosis treatment

	TB treatment initiations during inpatient stay N=450			
**Adverse event**	**SOC (N=182) n (%)**	**Intervention (N=268) n (%)**	**Malawi (N=185) n (%)**	**South Africa (N=265) n (%)**
Any side effects[a]	96 (52.7)	134 (50.0)	113 (61.1)	117 (44.2)
Nausea/Vomiting	52 (28.6)	62 (23.1)	44 (23.8)	70 (26.4)
Jaundice	9 (4.9)	10 (3.7)	12 (6.5)	7 (2.6)
Abnormal liver function tests	6 (3.3)	8 (3.0)	5 (2.7)	9 (3.4)
Itching or new skin rash without known cause	42 (23.1)	59 (22.0)	41 (22.2)	60 (22.6)
Numbness or pain of fingers or toes	60 (33.0)	85 (31.7)	71 (38.4)	74 (27.9)
ART stopped due to side-effects	1 (0.5)	7 (2.6)	8 (4.3)	0
TB treatment stopped due to side-effects	4 (2.2)	9 (3.4)	9 (4.9)	4 (1.5)

[a] Side-effects were recorded from the hospital medical records during inpatient stay.

Table 8: Tuberculosis drug resistance diagnosis

	TB diagnoses during inpatient stay (N=474)			
	**SOC (N=192) n (%)**	**Intervention (N=282) n (%)**	**Malawi (N=194) n (%)**	**South Africa (N=280) n (%)**
Rifampicin resistance[a]	6 (3.1)	5 (1.8)	2 (1.0)	9 (3.2)

[a] There was also one false positive sputum Xpert rifampicin resistant result in the intervention arm. All other Xpert rifampicin resistance results were also resistant on confirmatory testing on a repeat specimen. SOC indicates standard of care, ART antiretroviral therapy.

Table 9: Proportion of pre-specified subgroups who were clinically suspected of tuberculosis

**Subgroup**	**Baseline CD4 count <100**		**Baseline CD4 count ≥100**	
Clinically suspected of tuberculosis	**Hb <8 g/dL**	**Hb ≥8 g/dL**	**Hb <8 g/dL**	**Hb ≥8 g/dL**
Yes	132 (53%)	270 (55%)	111 (33%)	479 (33%)
No	119 (47%)	225 (45%)	222 (67%)	993 (67%)
Total	251	495	333	1472

23 patients are missing data on CD4 cell count, haemoglobin or clinically suspected tuberculosis. % are column percentages. Hb denotes haemoglobin.
